# Prevention of cognitive deficits in Alzheimer’s mouse model by elevating brain magnesium

**DOI:** 10.1186/1750-1326-7-S1-L24

**Published:** 2012-02-07

**Authors:** Guosong Liu

**Affiliations:** 1 School of Medicine, Tsinghua University, Beijing, China

## 

Memory functions decline with age, and severely deteriorate during Alzheimer’s disease (AD). Several studies suggest that dietary/environmental factors can reduce the prevalence of AD in humans. Magnesium is essential for maintaining normal body and brain functions. Here we show that increasing brain magnesium, using a newly developed magnesium compound (magnesium-L-threonate, MgT), prevents cognitive deficits and pathological changes in transgenic mice co-expressing familial AD-linked APP and PS1 variants that mimics the pathological and behavioral changes of human AD (AD mice). In intact mice, brain Mg content was found to be the lowest among all tissues tested; long-term MgT-treatment significantly elevated Mg levels in brain, and was associated with markedly improved cognitive function. In AD mice, learning and memory abilities are seriously impaired by 7 months, and completely deteriorate at 15 months. Strikingly, MgT-treatment was effective in preventing such severe learning and memory deterioration during the entire course of AD progression. At the cellular level, MgT-treatment reduced amyloid deposition in hippocampus and frontal cortex, and prevented synapse loss in the dentate gyrus (DG) and CA1 areas. The degree of memory improvement was strongly correlated with the protection of synapse loss, but did not correlate with the reduction of Aβ plaque. Thus, increased brain Mg might block Aβ-induced synaptic dysfunction. Since AD patients already have Mg deficiency, increase in brain Mg may represent a new strategy for prevention and amelioration of AD.

